# Behavior Coding of Adolescent and Therapy Dog Interactions During a Social Stress Task

**DOI:** 10.3390/vetsci11120644

**Published:** 2024-12-12

**Authors:** Seana Dowling-Guyer, Katie Dabney, Elizabeth A. R. Robertson, Megan K. Mueller

**Affiliations:** Center for Animals and Public Policy, Cummings School of Veterinary Medicine, Tufts University, 200 Westboro Rd., North Grafton, MA 01536, USA; seana.dowling_guyer@tufts.edu (S.D.-G.); earr@comcast.net (E.A.R.R.)

**Keywords:** animal-assisted interventions, dog behavior, human behavior, anxiety, stress

## Abstract

Youth mental health interventions incorporating trained therapy animals are increasingly popular, but more research is needed to understand the specific interactive behaviors between participants and therapy dogs. Understanding the role of these interactive behaviors is important for supporting both intervention efficacy and animal welfare and well-being. The goal of this study was to develop ethograms to assess interactive behaviors (including both affiliative and stress-related behaviors) of participants and therapy dogs during a social stress task, explore the relationship between human and dog behaviors, and assess how these behaviors may vary between experimental conditions with varying levels of physical contact with the therapy dog. Using video data from a previous experimental study (*n* = 50 human–therapy dog interactions, *n* = 25 control group), we successfully developed behavioral ethograms that could be used with a high degree of interrater reliability. Results indicated differences between experimental conditions in dog and human behaviors based on whether participants were interacting with a live or a stuffed dog, and whether they were allowed to touch the dog. These findings suggest that physically interacting with a live dog may be an important feature of these interventions, with participants demonstrating increased positive behaviors such as laughing and smiling in these conditions. Dog behaviors also varied based on whether they were in the touching/petting condition of the study which could indicate reactions to the session and has potential welfare implications for the dogs. Future research should focus on identifying specific patterns of interactive behaviors between dogs and humans that predict anxiolytic outcomes.

## Simple Summary

Youth mental health services with therapy animals are growing, but more research is needed to understand how participants interact with therapy dogs. This study aimed to develop ethograms to assess youth and dog behaviors during a social stress task, examining how these behaviors change with different levels of physical contact. Results indicated that human and dog behaviors differed based on whether participants interacted with a live or stuffed dog, and if they could touch the dog. Interacting with a live dog was linked to more positive behaviors like smiling, while dog behaviors varied depending on physical contact.

## 1. Introduction

Therapeutic interventions and services incorporating trained therapy animals (often referred to as animal-assisted interventions or services; AAI/AAS) are popular as a strategy for supporting individuals experiencing anxiety. AAI/AAS typically involves partnering with trained therapy animals for the purpose of meeting therapeutic goals [1]. Research on AAI in mental health contexts has indicated that these interventions can be effective in supporting treatment outcomes [2,3,4,5], including anxiety reduction [6,7]. Interventions integrating animals also can promote motivation to engage in therapy [3]. Furthermore, there is evidence that contact with animals more generally, in a variety of therapeutic and non-therapeutic settings (including both AAI and pets in the home) can buffer cardiovascular responses to stress [8,9].

However, despite the promise of therapeutic approaches integrating animals, it is still unclear exactly how interacting with animals can promote anxiety reduction, and the degree to which these individual types of interactions might impact intervention efficacy. For example, there is a need for research that identifies the specific mechanisms of action by which interacting with animals can produce therapeutic outcomes. By nature, interactions with therapy animals can vary widely based on the individual human and animal participants. Yet, there is little knowledge about how specific interactions may produce anxiolytic effects, which is critical to designing evidence-based interventions. For example, since participants can ideally control the degree of engagement with the therapy animal (choosing to ignore the animal, look at the animal, talk to the animal, and/or touch the animal), as well as animals having choice in engaging with the human participants (choosing to physically interact, leaving the interaction), it is possible that the degree to which youth and animals choose to engage in the interaction may impact any potential benefits.

One hypothesized mechanism for the anxiety-reducing effects of contact with therapy animals is that the interaction with the animal provides social support. In other contexts, social support has been found to reduce the effects of laboratory-based stressors [10]. One early definition of stress was “a non-specific response of the body to a demand”. This response may be positive, in the case of eustress, which may stimulate motivation or focus, or it may be negative, distress, which may trigger physical and psychological ill-effects. Stressors are any stimuli that produce a stress reaction [11]. Humans rely on social support to evaluate stressors [12], and perceptions of the strength of one’s social relationships contributes significantly to how stress is perceived and managed. When interacting with an animal, a relationship is formed that can become a part of an individual’s representation of social resources and social support network. Strong attachment or connection to a pet has been associated with adaptive coping to stress [13], but this mechanism has not been assessed as extensively in interventions where relationships with the therapy animal may be transitory. There is little empirical evidence demonstrating how specific interactions relate to immediate or global anxiolytic responses.

Another possible mechanism for how specific interactions can reduce anxiety is physical touch. Prior research on interpersonal touch between humans [14] shows that touch can impact heart rate [15] and reduce pain perception [16]. Initial research on the role of human–dog physical contact suggests that this type of touch may be an important element of the interaction [17,18], and physical touching of a dog has been associated with lower cortisol in children during stressors [19]. Interestingly, one study found that contact with an animal was associated with lower anxiety than contact with a human friend [20], which further suggests that physical touch could be an important factor, beyond social support, for reducing anxiety. However, little is known about how much and what type of touch during these interactions is optimal, and what the temporal dynamics are for physical touch.

In addition, there has been relatively little research exploring how the behaviors of therapy animals can influence treatment efficacy within AAIs. Dogs have evolved with humans (for a review, see [21,22]) and research has demonstrated how attuned they can be to human presence and emotion [23,24,25,26]. For adolescents experiencing acute anxiety in a therapeutic setting, a therapy dog proactively initiating interaction may be the prompt an anxious teen needs to engage with the dog and potentially reduce their anxiety. In addition, a therapy dog’s affiliative or engaging behaviors could create a feedback loop that would sustain the interaction longer and ultimately increase the efficacy of the intervention. Yet there has been little research examining the role and specific behaviors of the dog in a therapeutic setting and how those may relate to treatment efficacy. Research examining the specific responses of therapy dogs working with anxious clients is needed to understand if, how, and when dogs may facilitate and improve therapeutic outcomes.

However, there also may be negative effects of therapy animal behavior on treatment efficacy as well as the ethical considerations of integrating animals into healthcare services. In recent years, there has been an increase in research focusing on negative stress in dogs in the context of therapeutic interventions, primarily measured via behavioral ethograms and salivary cortisol. While some studies have shown no substantive impact of participating in interventions on stress-linked behaviors in dogs [27,28,29,30], others have found evidence of subtle behavioral signs of stress [31]. The effects of stress-linked behaviors, even subtle ones, on both the therapy animal and the client, are not clear. Therapy dogs experiencing stress in a therapeutic setting may display stress-linked behaviors such as lip licking, avoiding the client, seeking comfort from their handler, gaze and touch avoidance, and, in more severe cases, trembling and hiding, among others [30,31]. A therapy dog who avoids gaze and contact with a participant may be ineffective at best, but such behaviors may, at worst, be interpreted by anxious youth as personally directed at them, further exacerbating their anxiety (in addition to the detrimental stress of the animal participants that these behaviors may reflect). Additional research is needed to examine specific stress-linked behaviors of animals displayed during AAIs, and adolescents’ reaction to them (in terms of both observable behavior and physiological responses), in order to understand the effect on the outcome of AAIs. Although therapy animal handlers are generally trained to identify stress in their animals, the degree to which even minor stress behaviors can impact the intervention is unknown. Understanding these behaviors in more detail is critical to supporting ethical AAI/AAS that respect both human and animal well-being.

As a first step towards understanding how specific human and dog behaviors may contribute to the efficacy of interventions incorporating dogs for anxiety, there is a need for developing behavioral coding tools that can be used in this specific setting. Previous ethogram work (e.g., [29]) has focused on dog behaviors; this study aims to extend this prior work by including human stress and affiliative behaviors and coding human and dog interaction in an integrative way. Using previously collected video data from an experimental protocol involving adolescents participating in a social stress task [32], the objective of this study is to (1) develop ethograms for assessing specific individual and interactive behaviors (including both affiliative and stress-related behaviors) of participants and therapy dogs during an anxiety-inducing situation, (2) assess if human and dog behaviors are correlated in this setting, and (3) explore how these behaviors may differ across conditions with regard to level of contact with the dog (no dog present, dog present but no physical contact with participant, dog present and participant allowed physical contact). This study will allow for a more nuanced understanding of how people and therapy dogs interact during a situation involving a stressor, which will set the stage for future research that can uncover how specific interactions may be best suited to producing anxiolytic effects and reducing the risk of stress in therapy dogs.

## 2. Materials and Methods

This study used existing data collected as part of a larger study [32] that tested the effects of interacting with a therapy dog for youth with social anxiety. The data used in this study include videos of experimental sessions where therapy dogs (along with their handlers) were interacting with adolescent participants. The experimental procedure is outlined below. The original experimental procedures were approved by the Tufts University Social Behavioral Educational Research Institutional Review Board (protocol #1702004) and the Institutional Animal Care and Use Committee (protocol #G2017-09). The original study was also registered on ClinicalTrials.gov (ID: NCT03249116). The video coding procedures for the analyses reported in this paper were additionally reviewed and approved by the Tufts University Social Behavioral Educational Research Institutional Review Board (protocol #1599), and use of video data was within the scope of the original study’s consent/assent. Preliminary results from an initial set of coding from this study were presented at a conference prior to publication [33].

### 2.1. Participants

Video data were collected during an experiment in which researchers enrolled 75 adolescents ages 13–17 (76% female; 77% pet owners) on a continuum of social anxiety. Individuals were screened for social anxiety using the Social Anxiety Scale for Adolescents (SAS-A; [34]). Parental consent and youth assent were obtained for the screening process. Based on existing research suggesting the assessment of social anxiety on a continuum [35], participants were stratified to select a sample of individuals who ranged across low (*n* = 18), mid (*n* = 22), and high social anxiety (*n* =35) using the cutoffs recommended by La Greca [34]. Exclusion criteria included fear of or allergy to dogs. Participants were randomized into one of three conditions: (1) social + physical interaction with a live, trained therapy dog (*n* = 25); (2) social interaction (no physical contact) with a live, trained therapy dog (*n* = 25); and (3) stuffed toy dog (active control condition; *n* = 25).

#### Therapy Dogs

All dog/handler teams were members of Tufts Paws for People and evaluated and registered through the Pet Partners^®^ therapy animal organization. Per the Pet Partners^®^ guidelines, all dogs were on a 6-foot leash for the duration of the experimental sessions. All animal handlers had completed a handler training course and their animals passed a rigorous evaluation (with re-evaluation every 2 years) to meet training, safety, and health standards. The therapy dogs were bathed/groomed 24 h prior to participating in the study, and the teams were covered by comprehensive liability insurance through Pet Partners^®^. The dogs were always accompanied by their handlers, who were trained to recognize stress and discomfort signals in their dogs (e.g., attempting to leave the room, repeated or excessive yawning and panting, turning away). Study procedures stipulated that if any dog was deemed by their handler or the research team as becoming stressed by the visit, the interaction would be discontinued, and an alternate team would be substituted for future participants; however, no instances of this occurred. In total, four dogs were used for this study, all <30 pounds in weight (*n* = 3 female; *n* = 1 male), ranging from 8 to 13 years.

### 2.2. Original Experimental Procedure

The primary task all participants completed was the Trier Social Stress Task for Children (TSST-C), which involves six distinct phases: baseline, anticipation, preparation, speech, mental math, and recovery [36]. The TSST-C has been used extensively as a robust and reliable method for inducing social stress, and variants have been used in conjunction with animal interaction (e.g., [20]). The TSST-C was originally validated for use in 10–14 year-old youth [36], but the protocol used in our study was a specific adaptation validated for use in adolescents up to 17 years old [37]. The TSST-C involves a public speaking task and a mental arithmetic performance challenge. Participants in all three conditions listened to an experimenter speak for several minutes about a therapy dog and saw a photo of the dog, in order to further control for the novelty effects of an animal stimulus and to provide a baseline rest period (20 min). After a baseline period, participants were told about the stress task (anticipation). Then, participants had a 5 min preparation period, after which they were asked to speak on an academic topic for 5 min (e.g., discuss a historical figure, give a plot summary and interpretation of a book of their choice). Participants were then asked to complete a serial subtraction mental arithmetic task for 5 min, with difficulty level adjusted for age. Finally, there was a 30 min recovery period.

In the control condition (1), participants listened to the verbal presentation but there was no animal present. Instead, there was a stuffed toy dog to serve as active control [19] and a person (to mirror the therapy dog handler). In both animal interaction conditions, one therapy dog and their handler accompanied the participant during all phases of the TSST-C. In the social interaction condition (2), participants were told that the dog would be present, and they were able to socially interact with it at any point during the experiment (i.e., talking), but they were not permitted to touch the dog. In the social + physical interaction condition (3), participants were told that the dog would be next to them, and they were encouraged to interact socially and touch the dog during the experiment.

**Figure 1:**
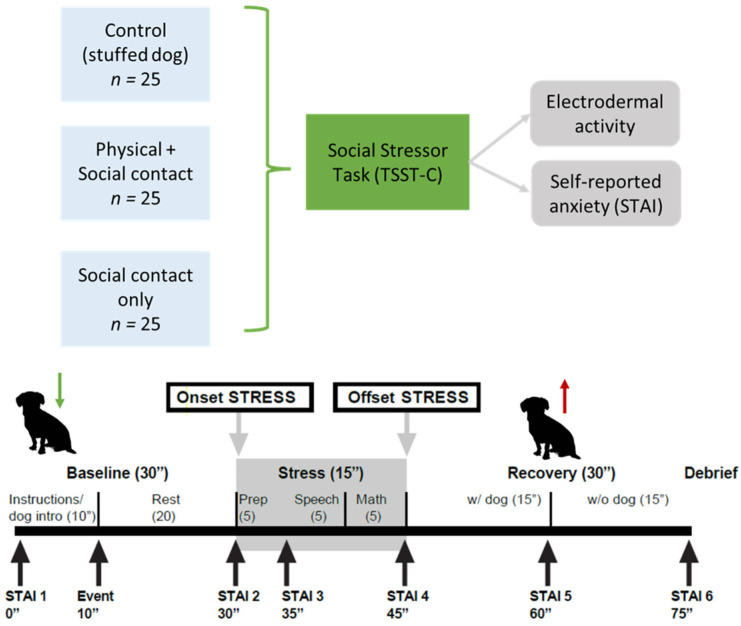
Original experimental conditions and study timeline/tasks.

Animal handlers were instructed to provide consistently minimal verbal contact, to reduce the confounding effects of handler variability and distraction from the TSST-C tasks. To simulate a typical AAI/S environment, the handler remained in proximity to the dog to monitor their behavior but did not interfere with the TSST-C tasks in any way. All sessions were videotaped in their entirety using two cameras at consistent angles to capture both participant and dog behavior. See Figure 1 for a diagram of experimental conditions and study timeline/tasks. 

### 2.3. Video Coding Procedure

To code the videos for dog and human stress and affiliative behaviors, we used Noldus Observer XT 14 software with advanced analysis and multiple media modules to analyze video files from the experiment involving adolescents and therapy animals. All sessions were videotaped from two angles to ensure high quality images of both the participants and the therapy dogs. Researchers used an ethogram to code social, physical, and verbal contact between participants and therapy dogs as well as independent movements and behaviors. Each video was coded using standardized coding manuals to train raters, code videos, and assess inter-rater reliability regularly throughout the coding process.

### 2.4. Measures

#### 2.4.1. Behavior Coding

We developed dog and human behavior ethograms for this study based on prior research on both dogs [27,31,38,39,40,41,42,43,44]) and humans [45,46,47,48], including past research on AAIs [27,31,49] and behavior coding within the TSST-C paradigm [50]. The ethograms were developed by reviewing prior literature and generating a list of behaviors associated with movement, stress/stress coping, and affiliative/contact seeking interactions, and specific operational definitions were created for each of these behaviors. Behaviors were coded as duration (in seconds) or count (frequency), depending on the nature of the behavior. The draft ethogram was reviewed by the research team and edited through an iterative process including testing with non-study videos. Next, the ethograms were tested on two study videos that were not used as part of the study analyses. The ethograms were further refined as part of this process, including adding and removing behaviors and creating more specific operational definitions for the behaviors until adequate inter-rater reliability could be reached. Behaviors with continued poor reliability after training were dropped from analyses.

Two coders rated the videos, and they achieved excellent interrater reliability for the final analytic behaviors (Intraclass correlation [ICCI] using a two-way random effects for consistency agreement model = .81 to 1.00, *M* = .95, for human behaviors in nine videos, ICC = .62 to 1.00, *M* = .93, for dog behaviors in 50 videos). One set of codes was used for analytical purposes.

#### 2.4.2. Adolescent Behaviors

Adolescent behaviors were coded in several broad categories (see Table 1 for full ethogram). Behaviors included different types of vocalizations, facial movements, eye activity, head position, and body movement (including stress-linked behaviors such as fidgeting and nail biting, as well as body positioning relative to the therapy dog). Behaviors that were related to the experimental task were coded (such as math or speech task), as well as the participant’s location in the room; however, these behaviors were not included in the analyses and so are not included in Table 1.

#### 2.4.3. Dog Behaviors

Similarly, dog behaviors were coded into categories related to locomotion (e.g., jumping, walking), body position (e.g., laying alert, sitting, location), tail position, body actions (including stress behaviors such as panting, whining), and dog behavior towards participant (e.g., head towards participant). See Table 2 for full ethogram.

### 2.5. Data Analysis

From the initial set of 26 human behaviors (Table 1) and 91 dog behaviors (Table 2) that were coded, one human and 36 dog behaviors were excluded from analysis as they were not exhibited by any participant/dog or were observed for less than one second (the latter behaviors tended to be coded inconsistently). One human and ten dog behaviors had poor interrater reliability (kappas < .60) or were not detectable due to audio quality or camera angles, so were excluded. Finally, 17 dog behaviors were excluded because they were cued by the handler or participant or directed toward the handler. Table 1 and Table 2 identify excluded behaviors. These exclusions left a total of 24 human behaviors and 28 dog behaviors included in the analysis.

SPSS v29 was used for all analyses. *p* values < .05 were considered significant. Descriptive statistics (mean, median, minimum, maximum) were calculated for the total sample and within each group. Given the significant departures of the behavioral data from normality, non-parametric tests were used. Related-samples Wilcoxon signed-rank tests compared differences in frequencies or durations of some behaviors, such as when participants spoke to the dog versus the handler or experimenter. Independent samples Kruskal–Wallis tests were calculated to detect significant differences in behavioral data by experimental group although independent samples Mann–Whitney U tests were used when just the two groups with a live dog were compared. Visual inspection revealed that the distributions of the behavioral data were not similar for all groups, so mean ranks of distributions were used for these statistical tests. For significant Kruskal–Wallis tests, post hoc pairwise comparisons adjusted with a Bonferroni correction for multiple comparisons were used to identify group differences. To aid in the interpretation of the statistical tests, means are reported with medians, although test results are based on mean ranks. Spearman’s Rho determined the correlation between human and dog behavioral data.

In order to reduce the large number of behaviors used in the analyses to a more manageable number, Principal Components Analyses (PCA) were separately conducted for human and dog behaviors. To determine the suitability of the data for PCA, correlations amongst the behavior variables were examined and any behaviors which did not correlate with at least one other behavior at ±.30 or higher were excluded from PCA. Additionally, behaviors with an anti-matrix correlation < .50 were excluded. The overall Kaiser–Meyer–Olkin (KMO) measures for the final human and dog behavior PCAs were > .70, considered suitable for factor analysis [51], and both PCAs generated significant Barlett’s test of sphericity (*p* < .05). The scree plot along with eigenvalues were used to determine the number of components. Varimax orthogonal rotations were used to help with interpretation. Upon review, any behaviors which loaded on two or more components at ±.40 or higher and were within ±.10 loading on the second component were excluded and the PCA was re-run. The final models were deemed interpretable, so the final component solutions were retained. Component scores were calculated by averaging the behavior variables with factor loadings of ±.40 or higher. Cronbach’s alpha was calculated for each component, which identified several components with low internal reliability; upon review, it was determined that these components were made up of both count and duration behaviors, which differed in the range of values. Behavior variables in the final models were then converted to z scores and the PCAs were rerun. Component scores and Cronbach’s alpha coefficients were recalculated using the standardized behavior variables. Cronbach’s alpha coefficients were adequate for the standardized component scores; therefore, the component scores based on the standardized behavior variables were used for subsequent analyses using the component scores.

## 3. Results

### 3.1. Participant Behavior

A total of 24 behaviors were observed for at least one second or at least one time in the human participants. Table 3 presents descriptive statistics for the duration (s) or number of times a behavior was observed by experimental condition. Fidgeting was observed the most, followed by participants’ orienting their head toward the dog. Eight behaviors (bite lip, close eyes, cross arms, cry, general scratch, sit on hands, talks to dog, talks to self) were seen infrequently enough to result in median durations or counts of 0. The most common stress-linked behaviors displayed by the human participants were fidgeting, moving their mouth or tongue internally, and playing with their hair, all with median durations of over 30 s. Repositioning also was a frequently displayed behavior that is linked to stress (Med = 87 times). Other less commonly seen stress-linked behaviors included touching their face, grimacing or displaying facial tension, yawning, biting their nails, biting their lips, crying, and scratching. Participants displayed orientation or affiliative behaviors toward the dog including positioning their head toward the dog, touching the dog (even when directed not to touch the dog based on experimental condition), and leaning toward the dog. Talking to the dog was observed infrequently (Table 3). Participants talked to the experimenter or handler longer than they talked to the dog (talking to dog vs. experimenter: *z* = −5.50, *p* < .001; talking to dog vs. handler: *z* = −7.06, *p* < .001), a difference that was seen even when the two conditions with a live dog were pooled (talking to dog vs. experimenter: *z* = −5.45, *p* < .001; talking to dog vs. handler: *z* = −5.44, *p* < .001).

**Figure 2:**
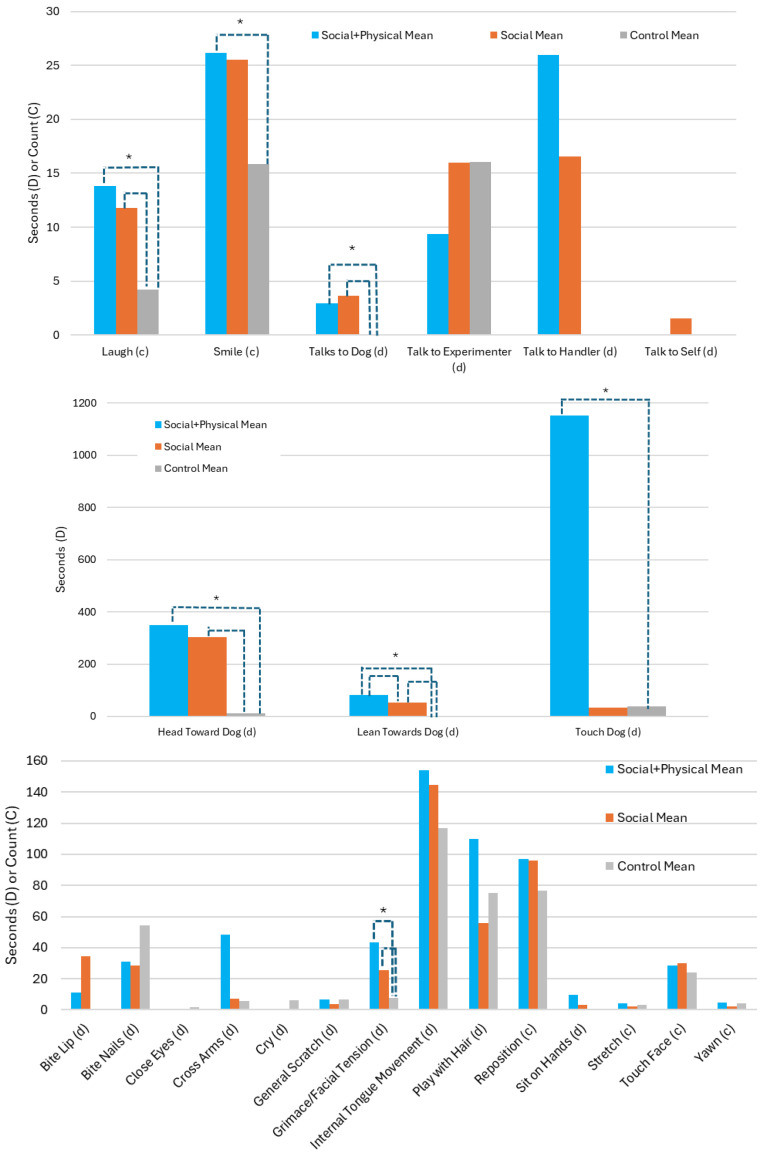
Human behaviors by experimental condition (*N* = 75). Note: * indicates difference of *p* < .05 between conditions based on mean rank testing, indicated by brackets.

Participants in the different experimental conditions displayed significant differences in the duration or number of times they exhibited seven behaviors (Table 3, Figure 2). Participants in the control group exhibited a grimace or facial tension for significantly less time than those in the social or social + physical groups, *H*(2) = 8.37, *p* = .015. When orienting toward the dog, participants in the control group faced toward the stuffed dog significantly less than participants in the social or social + physical groups faced the live dog, *H*(2) = 45.76, *p* < .001. Participants in the control group laughed significantly less than participants in the other two groups where live dogs were present, *H*(2) = 12.88, *p* = .002. Examining leaning toward the dog, the median duration for all groups differed significantly from all other groups, with participants in the social + physical group having a higher median duration, followed by participants in the social group, and then those in the control group, *H*(2) = 38.34, *p* < .001. Participants in the control group smiled significantly less than those in the social + physical group, *H*(2) = 9.12, *p* = .010. Finally, looking at interactions with the dog, participants in the control group talked to the dog significantly less than those in the other groups, *H*(2) = 25.11, *p* < .001. In terms of touching the dog, as expected due to the experimental conditions, participants in the social + physical group touched the therapy dog for significantly longer than participants in the other groups, *H*(2) = 50.98, *p* < .001. Interestingly, even though participants in the social group were instructed not to touch the therapy dog, some were observed doing just that, albeit significantly less than participants in the social + physical group. Participants in the control group were not given instructions about touching the stuffed dog, and some of them did do so. There were no other significant differences for participant behavior by experimental group, *p* > .05.

### 3.2. Therapy Dog Behavior

A total of 28 behaviors were observed for at least one second or at least one time in the four therapy dogs. Table 4 presents descriptive statistics for the duration (s) or number of times a behavior was observed by experimental condition. Laying down and laying alert (head up while laying down) occurred for the greatest total duration and shifting position was the most frequently counted behavior. Fourteen out of the 28 coded behaviors were seen infrequently enough to result in median durations or counts of 0, including common stress-linked behaviors such as panting, stretching, shaking off, or yawning (Table 4). The most commonly displayed behavior by the dogs related to the participant was positioning their head toward the participant, although the median duration for this behavior was just 26 s.

**Figure 3:**
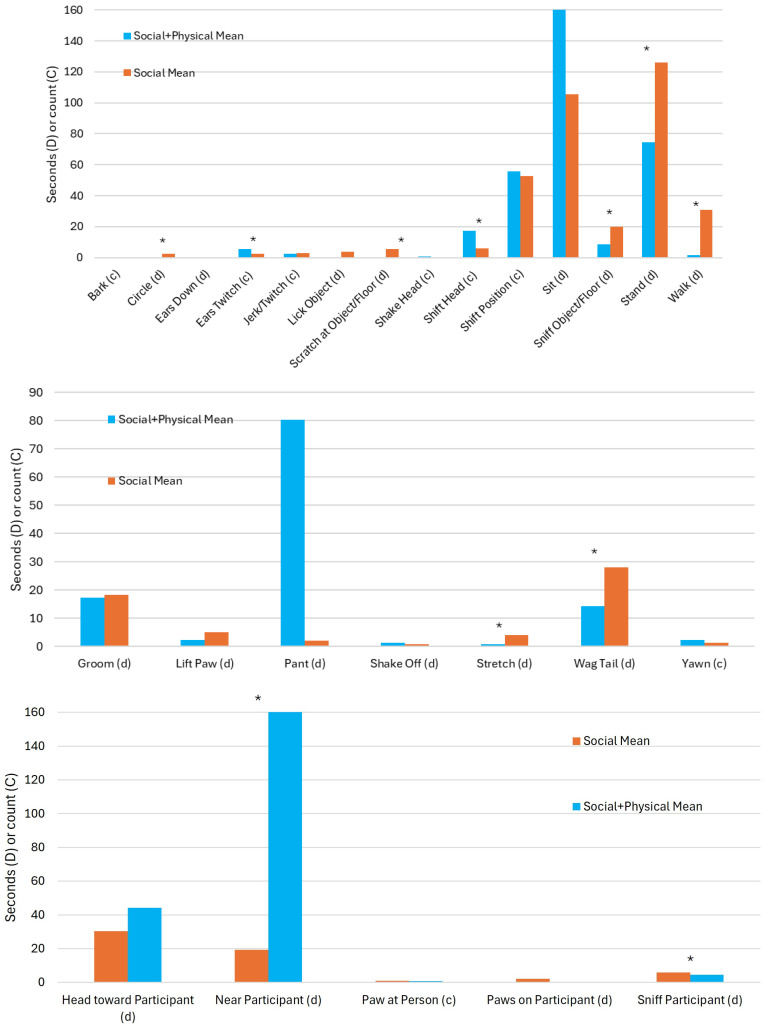
Dog behaviors by experimental condition (*n* = 50). Note: * indicates difference of *p* < .05 between conditions based on mean rank testing.

Therapy dogs in the two experimental conditions with live dogs displayed significant differences in the duration or number of times they exhibited 12 behaviors (Table 4, Figure 3). Dogs in the social + physical condition exhibited longer durations or higher counts of the following behaviors: ears twitching (*U* = 419.5, *p* = .036), laying down (*U* = 446.0, *p* = .010), and shifting their head (*U* = 499.0, *p* < .001). Dogs in the social condition displayed longer durations or higher counts of the following behaviors: circling (*U* = 225.0, *p* = .019), being near the participant (*U* = 203.0, *p* = .031), scratching an object or the floor (*U* = 225.0, *p* = .005), sniffing the participant (*U* = 211.0, *p* = .046), sniffing an object or the floor (*U* = 169.0, *p* = .005), standing (*U* = 124.0, *p* < .001), stretching (*U* = 224.0, *p* = .026), wagging tail (*U* = 176.5, *p* = .005), and walking (*U* = 20.0, *p* < .001). There were no other significant differences for dog behavior by experimental group, *p* > .05.

### 3.3. Participant and Dog Behavior Correlations

Table S1 presents the results of the correlation analysis for 672 behavior pairings for participants in the social + physical condition (*n* = 25), where both participants and therapy dogs were able to move freely. Table 5 summarizes the correlation results for participants in the social + physical condition (*n* = 25), presenting significant correlations of .30 or higher in grouped ranges, based on Spearman’s Rho coefficients (±.30–±.49, ±.50+). There were some potentially stress-linked behaviors in humans and dogs that were correlated, such as human grimace and dog circling, human arms crossed and dog barking, human biting lip and dog jerking/twitching, human playing with hair and dog licking the floor or an object, and human scratching and dog grooming. Interestingly, there were several instances of potential deflection behaviors on the part of the dog, such as a correlation between participants yawning and the dog not looking at the participant.

### 3.4. Participant Behavior Principal Components Analysis

A principal components analysis (PCA) was calculated using eight behaviors which met analysis requirements (correlation coefficients ±.30+ with at least one other behavior variable, anti-image correlation coefficients ≥.50, rotated factor loadings ±.40+ on one component or, if loading on two component, >±.10 difference between loadings). Behavioral data were converted to z scores, given the range in values between duration and count data which had resulted in low internal reliability. All PCA results and subsequent component scores are based on the standardized variables. The overall Kaiser–Meyer–Olkin (KMO) measure was .73, which Kaiser [51] classified as “middling”. Bartlett’s test of sphericity was statistically significant (*p* < .001), indicating that the data were likely suitable for PCA. 

PCA identified three components that had eigenvalues greater than one, explaining 34.4%, 21.8%, and 16.4% of the total variance in the rotated model, for a total of 72.6% (Table 6). Visual inspection of the scree plot supported the three-component solution, which was deemed interpretable. Cronbach’s alpha coefficients ranged from .91 (component 1) to .41 (component 3), with only component 1 achieving what is generally considered an acceptable value of .70 or higher [52,53].

### 3.5. Therapy Dog Behavior Principal Components Analysis

Using the same process as for participant behavior, a principal components analysis (PCA) was calculated using 10 standardized behavior variables which met analysis requirements. The overall Kaiser–Meyer–Olkin (KMO) measure was .73, which Kaiser [51] classified as “middling”. Bartlett’s test of sphericity was statistically significant (*p* < .001), indicating that the data were likely suitable for PCA. PCA identified three components that had eigenvalues greater than one, explaining 33.2%, 22.2%, and 12.1% of the total variance in the rotated model, for a total of 67.5% (Table 7). Visual inspection of the scree plot supported the three-component solution, which was deemed interpretable. Cronbach’s alpha coefficients ranged from .83 (component 1) to .23 (component 3), with components 1 and 2 achieving acceptable reliability.

### 3.6. Participant and Therapy Dog Behavior Component Scores by Experimental Group

Table 8 presents descriptive statistics for participant and dog behavior component scores based on standardized behavior variables by experimental group. There was a significant difference in participant component 3 scores by group, with participants in the social + physical group exhibiting those behaviors (smiling and touching the dog) more frequently than participants in the social or control groups, *H*(2) = 29.93, *p* < .001. Therapy dogs in the social group had significantly higher scores on dog component 1 (wagging, grooming, stretching, shifting position, and laying alert) than dogs in the social + physical group, *U* = 190.0, *p* = .017.

### 3.7. Correlations Between Participant and Therapy Dog Behavior Component Scores

When examining correlations for just the social + physical group, where both participant and dog were free to move around, there was one moderate negative but significant correlation between participant component 3 (smiling/touching the dog) and dog component 3 (being near to the participant and jerking/twitching) (Table 9).

## 4. Discussion

The goal of this study was to develop ethograms for assessing human and dog behaviors during a social stress task, and to evaluate if these behaviors were correlated and if they varied across experimental condition. We were able to successfully develop the ethograms with high interrater reliability and found that a number of the behaviors were correlated and varied across experimental condition. With additional research, this ethogram may be a useful tool for assessing participant and therapy dog interactive behaviors in an experimental setting. With future assessment, the tool may also have value in clinical or therapeutic settings as a method for monitoring dog welfare and human behavior.

The behavior coding results indicated some interesting differences between experimental conditions (social + physical involving physical contact with the therapy dog, social only with no touching of the therapy dog, and the control group with a stuffed dog). The participants spent more time facing towards the live therapy dog as well as more time smiling in the therapy dog conditions as compared to the control condition stuffed dog, which underscores the positive engagement that is associated with interacting with a live dog, as found in other prior research [49,54]. Similar to prior research [55], interacting with a dog may be associated with more positive affect, even during a stressful task. Somewhat unexpectedly, we found that participants grimaced and showed facial tension less frequently in control group. However, because this group also smiled less, it is possible that these facial behaviors are conflated, and this finding should be explored more in future research. 

Participants in the condition with physical contact also spent more time leaning towards the dog, which could indicate that physical touch is an important component of how participants interact with the dogs, aligning with prior research underscoring the role of touch in AAI/S [17]. There were also some differences in behaviors between dogs in the condition where they were being touched vs. not touched. For example, dogs exhibited more ear twitching and head shifting in the social + physical condition, although ear twitching was negatively correlated with the amount they were being touched. Dogs in the “social only” condition were more frequently close to participants, sniffing participants, and wagging their tail. It is possible that these dogs were trying to engage with the participants in the absence of the adolescents patting or touching them. Some dogs could find it mildly stressful to not interact with a nearby human, especially therapy dogs trained for that exact type of interaction. For example, prior research has found touch synchrony between handlers and dogs [56], a concept that could be explored between participants and therapy dogs. Further research should explore in more detail whether there are dog-specific factors or preferences for amount of touch and whether some dogs move more during physical touch than others and if/how/when they solicit touching, especially as these variations could contribute to therapeutic outcomes. For example, there may be personality preferences based on past experiences and training, or differences in desired interaction type based on the size of the dog (e.g., people may treat dogs of varying sizes differently [57]). It is important to note that dogs are individuals and are not interchangeable; thus, we need to approach research and practice on dog welfare in AAI/S with a personalized approach.

In addition to assessing behaviors across conditions, we also explored correlations between human and dog behaviors. We found some patterns of correlations between potentially stress-linked behaviors, such as participant grimacing and dog circling, participant’s arms crossed and dog barking, participant biting lip and dog jerking/twitching, participant playing with their hair and dog licking the floor or object, human scratching and dog grooming, and human yawning and dog facing away from the person. While some of these behaviors are not always linked to stress, it does suggest that there could be patterns where there are reciprocal stress behaviors. In particular, the co-occurrence of human scratching and dog grooming is an interesting finding that should be explored in future research to see if these patterns are replicated in other circumstances. Counterintuitively, we found few correlations between affiliative behaviors in the social + physical group, where both participant and dog could move freely and choose to interact or not. However, although they were allowed to interact or not, this interaction took place in a very structured experimental setting where the participant was completing various tasks, and therefore there was not as much opportunity for spontaneous interaction as in more naturalistic settings. Together, these findings all suggest the need for further research on the synchronization of dog and human behavior and the conditions under which synchrony takes place, as found with familiar pet dogs [58]. Research exploring the role of mutual gaze and petting on neural synchrony suggests that there are neural bases to the social interactions between people and dogs [59], though there is still much work to be done in identifying the complex mechanisms of social attachment in human–dog dyads [60]. Furthermore, a fruitful future direction of research would be to assess the timing of these behaviors and in which cases which species might be initiating behaviors. Initiation could be a key component of how a series of stress or affiliative behaviors might unfold.

Because the ethogram included a large range of behaviors, we employed PCA as a method of data reduction to attempt to create more interpretable factors that would be easier to use as a tool for assessment. We did find three components of behaviors for both humans and dogs. For human behaviors, one component included a group of “talking behaviors”, another component was related to fidgeting-type behaviors often associated with stress (biting nails, playing with hair, touching face), and another related to positive engagement (smiling and touching the dog). For dog behaviors, the conceptual nature of the components was a little bit less clear; the first component included tail wagging, grooming, stretching, shifting position, and laying alert, which seems to be a combination of friendly, relaxation, and mild stress behaviors. In addition, we did not assess the specific nature of the tail wagging behavior; tail wagging can indicate a wide range of emotions from friendliness, to alertness, to concern [61]. The second component included behaviors potentially responsive to the participant, such as pawing at the person, ear twitching, and facing their head towards the participant. The third component was only two factors—being near to the participant and jerking/twitching, which could potentially capture dogs who were not enjoying proximity to the participant, though this is a tenuous interpretation and needs to be explored in more detail. In addition, the reliability of the third components for both human and dog behaviors was poor, though they each contained only two items which is challenging for establishing good reliability, even with two items that are related to the same concept. Future research should assess if these components are correlated with outcomes such as self-reported anxiety or psychophysiological responses to stress in order to determine if they are useful as an assessment tool.

### 4.1. Limitations

Our study was limited by our relatively small sample of dogs and the lack of variability in many of the stress-linked behaviors. This sample of dogs was highly trained and experienced, and handlers were instructed to stop any interactions where stress behaviors occurred (although it should be noted that this did not happen at all during this study). Many common stress-linked behaviors, especially the more obvious/problematic behaviors, were not observed at all in this sample. This limitation highlights a methodological conundrum for studying therapy animal behavior in research settings. It is not ethical to integrate therapy dogs into settings when they are not prepared or they are demonstrating stress behaviors, and as researchers we have a responsibility to ensure that therapy dogs participating in our research are not, to the best of our knowledge, experiencing stress. However, it then makes it challenging to validate measurement tools for assessing dog stress in these settings. Nonetheless, we feel that measuring subtle stress behaviors is still worthwhile in moving towards a more nuanced understanding of how dogs indicate preferences for engaging in therapeutic services, especially given that owners and handlers often miss these subtle behavioral signs of stress [62]. Relatedly, we did not collect any biological measures of dog stress as part of this study, such as cortisol, glucocorticoid levels, or other indicators of the physical impacts of behavioral interactions. Future research with additional measures to triangulate with behavioral coding and to disentangle eustress from distress would be an important advancement.

Another limitation of this study is the specific experimental protocol. While this presented an excellent opportunity to validate behavior coding in a highly controlled and standardized setting, we recognize that these behaviors and patterns may not translate to all adolescent/dog interactions in therapeutic contexts. For example, our sample was strongly skewed towards participants identifying as female (as is a common limitation in human–animal interaction research [63]). There may be gender differences in some behaviors; for example, “playing with hair” was one of the most commonly observed stress-related behaviors, which may be a behavior that is more common in female participants, who often have longer hair. Future research with more balanced samples may wish to assess if there are gender differences in the type and frequency of coping behaviors as well as dog-directed behaviors. We encourage other researchers to use our ethogram if it is useful to them in other settings and with different populations (of both humans and dogs), which will enable expansion of validating the tool.

### 4.2. Conclusions and Future Directions

In conclusion, this study successfully developed ethograms for measuring therapy dog and adolescent behavior in the context of a stress task. We did not find evidence of any significant or severe stress behaviors in the therapy dogs. There were differences across experimental conditions in dog behaviors, suggesting that physical touch and freedom to interact physically with participants may be associated with patterns of dog behavior as they orient towards participants. Adolescents who were in the control group with no live dog laughed and smiled significantly less, suggesting that interactions with a live therapy dog may be associated with indicators of positive affect.

This study was successful in developing a behavioral coding tool that can be used to capture interactive behaviors between adolescents with anxiety and therapy dogs. Increasing the number of tools available to capture human and dog behaviors simultaneously will allow us to both better assess the efficacy of interventions involving therapy dogs as well as optimize welfare and well-being for our animal partners. Developing tools that allow us to assess the impact of specific human behaviors (for example, initiation of contact, human stress behaviors, human body movements) on therapy dogs is a necessary step for advancing animal welfare in AAI/S. By creating tools that are able to capture on a more precise timescale the precursors, consequences, and cascading effects of certain behavioral patterns, we can better work with practitioners and handlers to ensure dog well-being and active enjoyment when participating in therapeutic services. Considering the well-being of animals participating in AAI/S is of utmost importance when assessing evidence-based practice [64].

Future research should work to assess the association between these behaviors and therapeutic outcomes, such as anxiety and affect, and determine if the factors identified through the PCA are useful groupings for predicting outcomes. Similarly, as noted above, we should assess if these behaviors are correlated with other physiological measures of dog stress such as cortisol or oxytocin. In addition to assessing these outcomes, another useful area of exploration is assessing nuance in patterns of behaviors. For example, are there some situations where more or less of a particular behavior or set of behaviors is more or less beneficial? Does intensity and type of physical contact matter? Are there certain dogs with particular behavior types who have preferences for types of interactions with participants and what is the impact on therapeutic outcomes? Future research should examine a therapy dog’s possible synchrony as well as sensitivity to the participant’s affective state and stress levels, which suggest that a more active role for the therapy dog is possible, one that may be beneficial to AAI outcomes. There is an incredible amount of nuance in these behaviors, which means more sensitive and flexible measurement tools are needed, enabling further exploration of these complex questions.

## Figures and Tables

**Table 1:** Human behavior ethogram.

Behavior	Description of Behavior	Type (Duration (s)/Count)
**Vocalizations**		
Audible sounds ^2^	Stutters, ums, repetitive place holder noises	C
Laugh	Participant produces a laughing sound	C
Talk to experimenter	Participant engages in conversation, asks questions to experimenter, or responds to questions from experimenter	D
Talk to handler	Participant engages in conversation or asks questions to handler	D
Talk to self	Participant engages in self-oriented conversation	D
**Facial Movement**		
Bite lip	Lips are between teeth, outwards or inwards	D
Grimace/facial tension	Furrowed brow, squinted eyes, tight facial muscles, gritted teeth, clenched jaw, pursing lips	D
Internal tongue movement	Tongue movement inside the mouth, open or closed mouth, biting lip if teeth are not visible, lip licking, biting the tongue	D
Yawn	Participant yawns	C
Smile	Mouth is in an up-turned position	C
**Eye Activity**		
Cry	Tears are visible, face and mouth may quiver	D
Eyes closed	Eyes are closed for 2 s or longer	D
**Movement and Activity**		
Cross arms	Both arms are crossed in front of body	D
Bite nails	Biting nails, finger in mouth, fingertips on lips	D
General fidget	Foot bouncing, body swaying side to side, pronounced finger movement (entire finger moves, not just tip), finger/hand tapping, playing with clothing/wrist band. Movement must be 2 s or longer; stop code if there is a 2 s pause between instances, otherwise the code can continue running	D
General scratch	Itching or scratching body some place other than face	D
Play with hair	Twirling, running fingers through, twisting hair	D
Reposition	Includes body movements, arm shifts, big positional movements or arm place changes, arm/elbow moves or moves off arm rest, turning hand over (usually seen when arm is at rest on arm rest or lap)	C
Sit on Hands	Hands are under the thighs or bottom	D
Stretch	Shoulder shrug, double or single arm stretches, leg stretches, neck stretch (either side)	D
Touch face	Touching, stroking, scratching the face, rubbing hands on the chin, hand/hands holding the face, handing covering the mouth	C
**Location in Room**		
On chair ^3^	Subject is sitting on the chair	D
On couch ^3^	Subject is sitting on the couch	D
**Dog Orientation and Interaction**		
Head towards dog	Head is turned in direction of dog	D
Lean away from dog ^1^	Body tilts away from dog	D
Lean towards dog	Body tilts towards dog, within arm’s length	D
Talk to dog	Must be audible to coder and clear that the participant is speaking to dog vs. speaking in general	D
Touch dog	Participant initiates contact with hand on dog excluding accidental touching	D

Notes: ^1^ Behaviors not exhibited so excluded from analysis. ^2^ Excluded behaviors due to poor kappa (<.60) or poor visibility/audible on recordings. ^3^ Behaviors deemed irrelevant to analyses so excluded.

**Table 2:** Dog behavior ethogram.

Behavior	Description of Behavior	Type Duration(s)/Count
**Locomotion**		
Paws on handler ^1^	Dog jumps up and puts forelimbs onto the handler	D
Paws on inanimate object ^3^	Dog jumps up and puts forelimbs onto the object	D
Paws on participant	Dog jumps up and puts forelimbs onto the participant	D
Circle	Turning in one or more circles prior to sitting or lying down	D
Jump ^2^	Front two feet or all four paws off of the floor, either in the air or onto a wall or vertical surface; usually repetitive	C
Jump into/out of handler’s lap ^1^	Dog jumps off of the floor into the lap of the individual or off of lap to another surface	C
Jump into/out of participant’s lap ^2^	Dog jumps off of the floor into the lap of the individual or off of lap to another surface	C
Jump on/off couch/chair ^1^	Dog jumps on to or off of couch/chair	C
Lifted into/off chair/couch/bed ^1^	Handler lifts dog into the dog bed (may be on floor or chair)	C
Pace ^2^	Repeated locomotion around something or in fixed route (usually three times or greater)	D
Repetitive circling ^2^	Turning in multiple short circles while standing in the same location, not associated with circling prior to sitting or lying down or tail chasing	D
Run ^2^	Dog exhibits rapid locomotion from one location in the room to another with the movement forward movement of all four paws	D
Walk	Forward movement of all four paws resulting in shift of the whole body to a new position in the room	D
**Body Position**		
Crouch ^2^	Rapid and pronounced lowering of the posture	D
Cued behavior ^1^	Cued behavior initiated by handler’s verbal cue or hand signal	C
Lay alert	Dog is lying down with stomach in contact with surface but head is raised and/or not down, eyes open if visible	D
Lay down	Lying down, body in a ventral or lateral position, stomach in contact with a surface, head lowered or down	D
Lean away from participant ^2^	Dog leans away from the participant of the study, even if still in contact with participant, increasing distance without changing position	D
Lean on handler ^1^	Dog has physical contact with the handler, bearing weight against handler or leaning into hand	D
Lean on object ^2^	Dog has physical contact with an object, bearing weight against object	D
Lean on participant ^2^	Dog has physical contact with the participant, bearing weight against participant or leaning into hand	D
Rolling on back ^2^	Lying on ground with belly up lifting hind leg, or rolling onto back exposing ventral side, including half roll (fine tune—at least two legs in the air)	D
Shift within position	Whole body or individual body part movement usually when in lying down or sitting including readjustment from one position back to a similar form of that position under 2 s (Not including head movements)	C
Sit	Dog is sitting with front legs extended and hind legs flexed, stomach not in contact with surface	D
Stand	Dog is standing on all four paws, not moving	D
**Location in Room**		
In bed on floor ^3^	Dog is on bed/blanket/mat on the floor for 2 s or longer	D
In handler’s lap ^1^	Dog is in handler’s lap for 2 s or longer	D
In participant’s lap ^1^	Dog is in participant’s lap for 2 s or longer	D
Near handler ^1^	Close to dog handler, some part of dog is within 1.5–2 ft of the handler	D
Near participant	Close to research participant, some part of dog is within 1.5–2 ft of the participant	D
On chair/couch ^1^	On chair or couch near participant or handler (may or may not be on bed) for 2 s or longer	D
**Tail Position**		
Chase tail ^2^	Focuses on tail and repeatedly follows, trying to bite or lick tail, generally circling to follow tail	D
Tail tucked ^2^	Tail held still and tightly between hind legs, may be curled under genital area or ventral side	D
Tail up ^2^	Tail is erect, perpendicular to the body	D
Wag tail	Repetitive side-to-side movements of the tail; tail generally moves perpendicular to the dog’s body	D
**Body Actions**		
Tremble/cower ^2^	Shaking of the body or shivering, visible shaking while dog is still or cowering, all or part of the body shaking	D
Jerk/twitch	Sudden, quick movement with body/limb/skin/tail (usually the tip of the tail moves, movement not from the base of the tail), possibly involuntary movement	D
Lift paw	Forepaw is lifted into a position of approximately 45 degrees; dog is standing/sitting and lifts one paw	D
Paw at person	Touches person with paw, and moves paw in the area around the receiver of the action	C
Piloerect ^2^	Raising of hair on back anywhere from neck to tail	D
Roll over ^2^	Dog rolls completely over, from abdomen being ventral to dorsal to ventral again	D
Scratch at object/floor	Dog scrapes front claws at object or spot on floor	D
Shake off	Shaking of whole body, more overall movement than shake head	D
Stretch	Dog is extending/stretching a part of or whole body	D
**Head Actions**		
Ears back ^2^	Ears are back, for 0.5 s or more	D
Ears down	Ears are tucked down in a non-neutral position, for 0.5 s or more	D
Ears up ^3^	Ears are up and alert, for 0.5 s or more	D
Ears twitch	Sudden, quick non-directional movement of one or both ears	C
Eyes closed ^2^	Eyes closed for 2 s or more	D
Jerk head ^3^	A quick movement of the head, less than 2 s, that does not involve other body parts, and is not only ears	C
Look away ^2^	Dog was looking at participant/handler and then looked away, deliberate action	D
Nudge participant ^2^	Dog pushes part of participant with nose muzzle or head	D
Quick look ^3^	Looking somewhere else (not at a person) for less than 2 s	C
Repeatedly moving head ^2^	Changing head position continuously for 2 s or more without directly looking at a person/place	D
Shake head	Shaking head as if wet, but only head, not a full body shake	C
Shift head	A movement of the head that does not involve other body parts, is not only ears, is more purposeful than a jerk, does not include body movement	C
Side eye ^2^	Dog is looking out of the corner of eye	D
Sneeze ^2^	Sneezing	C
Sniff handler ^1^	Dog smells handler, determined by active nose twitching and rapid inhalation	D
Sniff object/floor	Dog smells object, determined by active nose twitching and rapid inhalation	D
Sniff participant	Dog smells participant, determined by active nose twitching and rapid inhalation	D
Tilt head ^2^	Entire head quickly oriented laterally and held stationary for at least 1 s; head tilted to the side, gesture may be done in response to a stimulus or due to dog looking at an object/person	C
**Head Actions (Gaze)**		
Head towards back of room ^3^	Dog is focused on the back of the room, by staring/gazing at back of the room for 2 s or longer	D
Head towards experimenter ^3^	Dog is focused on experimenter, by gazing/staring at experimenter	D
Head towards floor ^3^	Dog is focused on floor, by gazing/staring at floor for 2 s or longer	D
Head towards front of room ^3^	Dog is focused on the front of the room, by staring/gazing at front of the room for 2 s or longer	D
Head towards handler ^1^	Dog is focused on handler, by gazing/staring at handler	D
Head towards participant	Dog is focused on participant, by gazing/staring at participant	D
**Mouth Actions**		
Bite object ^2^	Contact by teeth to skin or clothing with intention to threaten or harm	D
Bite participant ^2^	Contact by teeth to skin or clothing with intention to threaten or harm	D
Drool/salivation ^2^	Dog has saliva hanging from the mouth or emanating from the mouth	D
Lick handler ^1^	Tongue extends to touch handler before retracting into mouth	D
Lick lip/nose ^2^	Tongue licking lips or nose, slowly or in rapid succession; dog is snout licking, tongue visible	C
Lick object	Tongue extends to touch object before retracting into mouth	D
Lick participant ^2^	Tongue extends to touch participant before retracting into mouth	D
Lift lip ^2^	Vertical retraction of lips, pulling upper lips back to show full teeth, usually paired with growling	C
Mouth open ^2^	Mouth is open, not panting or vocalizing	D
Mouthing ^2^	Teeth contact skin of participant with no break	D
Open/close mouth rapidly ^3^	Sequential opening and closing of mouth, within a timeframe of 2 s or less; not a lip lick or pant behavior	C
Pant	An increased frequency of inhalation and exhalation combined with opening of the mouth; tongue exposed with audible/or observable breathing; try to watch the abdomen	D
Snap at person ^2^	Teeth snap in air towards person and do not touch skin	C
Yawn	Opens mouth widely and inhales	C
**Vocalization**		
Bark	Short vocalization of low frequency, sometimes cyclical; rough sound often repeated in quick succession	C
Howl ^2^	Long, drawn out voice vocalization of high amplitude	D
Whine ^2^	Soft, high-pitched vocalizations; a cyclic vocalization; sustained whimper	D
Yelp ^2^	High-pitched vocalization usually occurring in response to a painful or unpleasant stimulus	C
**Maintenance**		
Groom	Dog bites/nibbles, licks, sniffs, scratches self or performs other non-specific maintenance behaviors	D
**Dog Handler Actions**		
Handler gives command/cue ^1^	Handler issues a command or cue, verbally and/or visually, for the dog to behave in a certain way or perform a behavior	C
Handler gives dog treat ^1^	Handler gives dog a treat, may be part of a command/cue but could also be without an associated behavior	C
Handler speaks to dog ^1^	Must be audible to coder and clear that the participant is speaking to dog vs. speaking in general	D
Handler touches dog ^1^	Handler initiates contact with hand on dog excluding accidental touching	D

Notes: ^1^ Excluded behaviors as directed or influenced by handler or participant. ^2^ Behaviors not exhibited or observed < 1 s so excluded from analysis. ^3^ Excluded behaviors due to poor kappa (<.60) or poor visibility/audible on recordings.

**Table 3:** Descriptive statistics of observed human participant behavior (75 trials) by experimental group (25 trials each).

Behavior	Total					Social + Physical					Social					Control				
	Mean	Med	Min	Max	N	Mean	Med	Min	Max	N	Mean	Med	Min	Max	N	Mean	Med	Min	Max	N
Bite Lip (d)	15.3	0.0	0.0	787.2	75	11.0	0.0	0.0	263.0	25	34.7	0.0	0.0	787.2	25	0.2	0.0	0.0	5.6	25
Bite Nails (d)	37.9	1.3	0.0	466.4	75	30.9	3.8	0.0	340.5	25	28.3	1.2	0.0	392.3	25	54.4	0.0	0.0	466.4	25
Close Eyes (d)	1.0	0.0	0.0	22.9	75	0.7	0.0	0.0	9.1	25	0.5	0.0	0.0	10.2	25	1.8	0.0	0.0	22.9	25
Cross Arms (d)	20.4	0.0	0.0	770.2	75	48.4	0.0	0.0	770.2	25	7.4	0.0	0.0	96.0	25	5.5	0.0	0.0	111.6	25
Cry (d)	2.1	0.0	0.0	149.1	75	0.0	0.0	0.0	0.0	25	0.0	0.0	0.0	0.0	25	6.4	0.0	0.0	149.1	25
General Fidget (d)	759.4	706.4	0.0	2555.4	75	789.2	724.9	0.0	2555.4	25	859.7	760.9	0.0	1757.4	25	629.2	520.9	15.3	1700.6	25
General Scratch (d)	5.7	0.0	0.0	101.2	75	6.7	3.1	0.0	35.5	25	3.6	0.0	0.0	32.1	25	6.6	0.0	0.0	101.2	25
Grimace/Facial Tension (d) *	25.6	5.3	0.0	548.9	75	43.3	11.0 ^a^	0.0	548.9	25	25.8	7.9a ^a^	0.0	233.0	25	7.7	1.7 ^b^	0.0	61.4	25
Head Toward Dog (d) *	221.8	135.6	0.0	3085.0	75	350.4	343.3 ^a^	0.0	1176.3	25	302.5	169.9 ^a^	0.0	3085.0	25	12.6	10.2 ^b^	2.2	29.9	25
Internal Tongue Movement (d)	138.5	53.5	0.0	1441.7	75	154.2	68.9	0.0	867.7	25	144.6	44.0	0.0	656.2	25	116.7	43.9	0.4	1441.7	25
Laugh (c) *	9.9	5.0	0.0	57.0	75	13.8	10.0 ^a^	0.0	57.0	25	11.8	8.0 ^a^	0.0	47.0	25	4.2	1.0 ^b^	0.0	24.0	25
Lean Towards Dog (d) *	45.6	3.2	0.0	1097.5	75	82.8	15.1 ^a^	0.0	1097.5	25	54.1	10.6^b^	0.0	329.9	25	0.0	0.0 ^c^	0.0	0.0	25
Play with Hair (d)	80.2	33.9	0.0	801.6	75	109.7	38.8	0.0	801.6	25	55.9	36.0	0.0	378.0	25	75.0	26.4	0.0	697.1	25
Reposition (c)	89.9	87.0	0.0	214.0	75	97.1	95.0	0.0	212.0	25	96.0	100.0	0.0	214.0	25	76.5	67.0	23.0	162.0	25
Sit on Hands (d)	4.3	0.0	0.0	196.2	75	9.5	0.0	0.0	196.2	25	3.4	0.0	0.0	56.1	25	0.0	0.0	0.0	0.0	25
Smile (c) *	22.5	19.0	0.0	69.0	75	26.2	22.0 ^a^	0.0	62.0	25	25.5	22.0 ^a,b^	0.0	69.0	25	15.9	17.0 ^b^	1.0	33.0	25
Stretch (c)	3.3	2.0	0.0	40.0	75	4.2	2.0	0.0	40.0	25	2.3	1.0	0.0	14.0	25	3.4	2.0	0.0	21.0	25
Talk to Dog (d) *	2.2	0.0	0.0	41.0	75	3.0	1.4 ^a^	0.0	25.6	25	3.7	1.1 ^a^	0.0	41.0	25	0.0	0.0 ^b^	0.0	0.0	25
Talk to Experimenter (d)	13.8	8.8	0.0	154.3	75	9.4	7.0	0.0	23.9	25	16.0	7.6	0.0	154.3	25	16.1	10.3	2.5	51.3	25
Talk to Handler (d)	14.2	2.2	0.0	117.5	75	26.0	17.9	0.0	117.5	25	16.5	4.2	0.0	103.9	25	0.1	0.0	0.0	2.0	25
Talk to Self (d)	0.5	0.0	0.0	37.9	75	0.1	0.0	0.0	2.8	25	1.5	0.0	0.0	37.9	25	0.0	0.0	0.0	0.0	25
Touch Dog (d) *	407.8	20.6	0.0	2856.8	75	1153.3	998.7 ^a^	47.4	2856.8	25	32.6	9.6^b^	0.0	268.3	25	37.5	0.0^b^	0.0	380.9	25
Touch Face (c)	27.5	26.0	0.0	93.0	75	28.6	29.0	0.0	65.0	25	29.8	21.0	0.0	93.0	25	24.2	18.0	1.0	63.0	25
Yawn (c)	3.6	2.0	0.0	16.0	75	4.8	4.0	0.0	16.0	25	2.1	2.0	0.0	10.0	25	4.0	1.0	0.0	16.0	25

Notes: Medians with different superscript letters significantly different at *p* < .05. * *p* < .05.

**Table 4:** Descriptive statistics of observed therapy dog behavior (50 trials total) by experimental group (25 trials each).

Behavior	Total					Social+ Physical					Social				
	Mean	Med	Min	Max	N	Mean	Med	Min	Max	N	Mean	Med	Min	Max	N
Bark (c)	0.1	0.0	0.0	2.0	50	0.1	0.0	0.0	2.0	25	0.1	0.0	0.0	2.0	25
Circle (d) *	1.4	0.0	0.0	17.3	50	0.5	0.0	0.0	7.6	25	2.3	0.0	0.0	17.3	25
Ears Down (d)	0.1	0.0	0.0	4.5	50	0.2	0.0	0.0	4.5	25	0.0	0.0	0.0	0.0	25
Ears Twitch (c) *	4.1	3.0	0.0	30.0	50	5.5	3.0	0.0	30.0	25	2.6	2.0	0.0	14.0	25
Groom (d)	17.9	0.0	0.0	195.8	50	17.4	0.0	0.0	195.8	25	18.4	3.5	0.0	119.6	25
Head toward Participant (d)	37.2	26.3	0.0	210.1	50	44.1	27.2	7.4	210.1	25	30.3	17.7	0.0	104.9	25
Jerk/Twitch (c)	2.5	0.0	0.0	20.0	50	2.3	0.0	0.0	20.0	25	2.7	0.0	0.0	18.0	25
Lay Alert (d)	556.4	296.0	64.6	2989.6	50	327.7	273.4	72.9	968.7	25	785.2	352.7	64.6	2989.6	25
Lay Down (d) *	2348.5	2749.2	0.0	3032.0	50	2609.4	2807.4	1180.1	3032.0	25	2087.6	2455.1	0.0	2968.5	25
Lick Object (d)	2.1	0.0	0.0	96.2	50	0.2	0.0	0.0	4.2	25	3.9	0.0	0.0	96.2	25
Lift Paw (d)	3.8	0.0	0.0	99.3	50	2.3	0.0	0.0	49.4	25	5.2	0.0	0.0	99.3	25
Near Participant (d) *	189.7	6.0	0.0	2871.7	50	360.2	0.0	0.0	2871.7	25	19.2	14.8	0.0	98.0	25
Pant (d)	41.2	0.0	0.0	1182.7	50	80.3	0.0	0.0	1182.7	25	2.0	0.0	0.0	27.5	25
Paw at Person (c)	0.8	0.0	0.0	10.0	50	0.7	0.0	0.0	10.0	25	1.0	0.0	0.0	10.0	25
Paws on Participant (d)	1.2	0.0	0.0	22.1	50	0.2	0.0	0.0	4.2	25	2.2	0.0	0.0	22.1	25
Scratch at Object/Floor (d) *	2.7	0.0	0.0	43.2	50	0.0	0.0	0.0	0.0	25	5.5	0.0	0.0	43.2	25
Shake Head (c)	0.5	0.0	0.0	4.0	50	0.7	0.0	0.0	4.0	25	0.4	0.0	0.0	3.0	25
Shake Off (d)	1.1	0.0	0.0	14.0	50	1.3	0.0	0.0	14.0	25	1.0	0.0	0.0	4.4	25
Shift Head (c) *	11.7	7.5	0.0	81.0	50	17.4	14.0	0.0	81.0	25	6.1	5.0	0.0	31.0	25
Shift Position (c)	54.2	40.5	1.0	212.0	50	55.8	40.0	19.0	212.0	25	52.6	41.0	1.0	121.0	25
Sit (d)	136.7	25.9	0.0	1198.5	50	167.8	8.7	0.0	1198.5	25	105.7	35.2	0.0	542.2	25
Sniff Object/Floor (d) *	14.3	4.8	0.0	96.6	50	8.6	2.4	0.0	96.6	25	20.1	11.3	0.0	80.9	25
Sniff Participant (d) *	5.2	2.0	0.0	46.1	50	4.5	1.0	0.0	46.1	25	5.9	3.6	0.0	28.5	25
Stand (d) *	100.4	35.5	0.0	965.0	50	74.5	2.9	0.0	947.4	25	126.2	64.8	11.3	965.0	25
Stretch (d) *	2.5	0.0	0.0	23.8	50	1.0	0.0	0.0	10.3	25	4.1	0.0	0.0	23.8	25
Wag Tail (d) *	21.2	0.8	0.0	201.4	50	14.3	0.0	0.0	201.4	25	28.0	15.2	0.0	124.8	25
Walk (d) *	16.1	4.2	0.0	101.6	50	1.4	0.0	0.0	16.0	25	30.7	26.4	1.8	101.6	25
Yawn (c)	1.9	1.0	0.0	11.0	50	2.5	2.0	0.0	11.0	25	1.4	1.0	0.0	6.0	25

Notes: * *p* < .05.

**Table 5:** Significant correlations between participant and therapy dog behavior, social + physical group only (*n* = 25).

Spearman’s Rho Coefficient Ranges for Human–Dog Behavior Pairings	
±.30–±.49	±.50–±.79
Human bite lip–Dog jerk/twitch Human cross arms–Dog bark Human cross arms–Dog lay alert Human cross arms–Dog lay down (neg) Human cross arms–Dog stand Human cross arms–Dog wag tail Human general scratch–Dog lay alert Human general scratch–Dog shift position Human general scratch–Dog sniff floor/object Human general scratch–Dog wag tail Human grimace–Dog circle Human grimace–Dog near participant (neg) Human internal tongue movement–Dog shake head (neg) Human laugh–Dog shake off (neg) Human laugh–Dog shift within position (neg) Human laugh–Dog sniff participant (neg) Human laugh–Dog stand (neg) Human play with hair–Dog lick floor/object Human smile–Dog near participant (neg) Human talk to dog–Dog lay alert Human talk to experimenter–Dog lay alert Human talk to experimenter–Dog lay down (neg) Human talk to experimenter–Dog shake head (neg) Human talk to handler–Dog head to participant (neg) Human talk to handler–Dog paw at person (neg) Human talk to handler–Dog sit (neg) Human talk to handler–Dog walk (neg) Human touch dog–Dog paw at person (neg) Human touch dog–Dog shift head Human touch dog–Dog walk (neg) Human yawn–Dog head to participant (neg) Human yawn–Dog near participant (neg)	Human cross arms–Dog stretch Human general scratch–Dog groom Human internal tongue movement–Dog sniff object/floor Human laugh–Dog near participant (neg) Human laugh–Dog walk (neg) Human talk to handler–Dog ears twitch (neg) Human talk to handler–Dog near participant (neg) Human talk to handler–Dog shift position (neg) Human talk to handler–Dog sit (neg) Human touch dog–Dog ear twitch (neg) Human touch dog–Dog near participant (neg) Human touch dog–Dog stand (neg) Human yawn–Dog walk (neg)

Notes: Neg = negative correlation. Groupings by Spearman’s Rho coefficients for human–dog behavior analysis; significant correlations (*p* < .05) only. A total of 672 correlations were examined. Table S1 presents the full correlation table.

**Table 6:** Rotated component matrix for PCA of observed participant standardized behaviors (*N* = 75).

Standardized Behavior	Component		
	1	2	3
Talk to Self (d)	0.93	0.07	−0.01
Talk to Experimenter (d)	0.89	0.23	−0.06
Talk to Dog (d)	0.87	−0.01	0.25
Bite Nails (d)	−0.12	0.85	0.10
Play with Hair (d)	0.19	0.77	−0.11
Touch Face (c)	0.46	0.61	0.09
Touch Dog (d)	−0.10	−0.02	0.82
Smile (c)	0.24	0.06	0.75
Eigenvalue	3.1	1.5	1.3
% of variance	34.4%	21.8%	16.4%
Cronbach’s alpha	0.91	0.65	0.41

Notes: Varimax rotation of standardized behavior data. Shading represents items that comprise each component.

**Table 7:** Rotated component matrix for PCA of observed therapy dog standardized behaviors (*n* = 50).

Standardized Behavior	Component		
	1	2	3
Wag (d)	0.91	0.01	0.01
Groom (d)	0.89	−0.06	−0.05
Stretch (d)	0.84	−0.19	0.001
Shift Position (c)	0.77	0.46	0.12
Lay Alert (d)	0.41	−0.001	−0.35
Paw at Person (c)	−0.05	0.84	−0.12
Ear Twitch (c)	0.05	0.77	0.06
Head toward Participant (d)	−0.08	0.73	0.30
Near Participant (d)	−0.08	0.29	0.79
Jerk/Twitch (c)	0.48	−0.26	0.58
Eigenvalue	3.3	2.2	1.2
% of variance	33.2%	22.2%	12.1%
Cronbach’s alpha	0.83	0.71	0.23

Notes: Varimax rotation of standardized behavior data. Shading represents items that comprise each component.

**Table 8:** Descriptive statistics of standardized participant behavior components by experimental group.

Behavior Component	Total (*N* = 75)				Social + Physical (*n* = 25)				Social (*n* = 25)				Control (*n* = 25)			
	M	Med	Min	Max	M	Med	Min	Max	M	Med	Min	Max	M	Med	Min	Max
**Participant**																
Component 1	0.00	−0.57	−1.20	22.22	−0.19	−0.45	−1.20	4.38	0.58	−0.56	−1.20	22.22	−0.39	−0.68	−1.07	1.39
Component 2	0.00	−0.68	−2.30	9.32	0.17	−0.58	−2.30	7.57	−0.15	−0.88	−2.30	5.31	−0.02	−1.00	−2.21	9.32
Component 3 *	0.00	−0.42	−2.20	6.31	1.30	1.09 ^a^	−0.86	6.31	−0.30	−0.60 ^b^	−2.20	2.87	−1.00	−0.90 ^b^	−2.16	0.21
**Therapy Dog**	**Total (*n* = 50)**				**Social + Physical (*n* = 25)**				**Social (*n* = 25)**							
Component 1 *	0.00	−0.35	−0.67	3.02	−0.16	−0.46 ^a^	−0.60	3.02	0.16	0.02 ^b^	−0.67	1.94	--	--	--	--
Component 2	0.00	−0.29	−0.67	3.51	0.12	−0.31	−0.53	3.51	−0.12	−0.26	−0.67	1.57	--	--	--	--
Component 3	0.00	−0.38	−0.41	3.33	0.13	−0.31	−0.41	3.33	−0.13	−0.38	−0.41	1.41	--	--	--	--

Notes: Medians with different superscript letters significantly different at *p* < .05. * *p* < .05.

**Table 9:** Participant and therapy dog behavior component correlations, social + physical group only (*n* = 25).

Behavior Components		Participants			Therapy Dogs		
		Component 1	Component 2	Component 3	Component 1	Component 2	Component 3
**Participants**	Component 1	1.00	0.01	0.49 *	0.28	0.20	−0.05
	Component 2		1.00	−0.05	−0.06	0.10	0.12
	Component 3			1.000	−0.08	−0.37	−0.61 *
**Therapy Dogs**	Component 1				1.00	0.50 *	0.38
	Component 2					1.00	0.33
	Component 3						1.00

Notes: Spearman’s Rho coefficients for standardized behavior data. * *p* < .05.

## Data Availability

The data from the original experimental study are openly available in the Open Science Framework at https://osf.io/w7k8p/?view_only=965f3d7390d7434895216fe8b88a2160. (accessed on 27 October 2024) Video data files are not publicly available due to the identifying nature of the videos (i.e., participant faces). Video code data are available upon request from the corresponding author.

## Supplementary Materials

The following supporting information can be downloaded at: https://www.mdpi.com/article/10.3390/vetsci11120644/s1, Table S1: Correlations Between Participant and Therapy Dog Behaviors, Social + Physical Group (*N* = 25).
